# Quality of services offered to women with female genital mutilation across health facilities in a Kenyan County

**DOI:** 10.1186/s12913-022-07979-3

**Published:** 2022-05-07

**Authors:** Samuel Kimani, Chantalle Okondo, Jacinta Muteshi-Strachan, Jaldesa Guyo

**Affiliations:** 1 University of Nairobi, P.O Box 19676-00202, Nairobi, Kenya; 2 Africa Coordinating Centre for the Abandonment of Female Genital Mutilation /Cutting (ACCAF), P.O Box 19676-00202, Nairobi, Kenya; 3 School of Nursing sciences, Kenyatta National Hospital, Po Box 19676-00202, Nairobi, Kenya; 4 Population Council-Kenya, PO Box 17643-00500, Nairobi, Kenya; 5 Options Consultancy Services Limited, St Magnus House, 3 Lower Thames St, London, EC3R 6HD UK

**Keywords:** Female genital mutilation, Quality of services, Health system response, Prevention, Care services, West Pokot, Kenya

## Abstract

**Background:**

Female genital mutilation (FGM) curtails women’s health, human rights and development. Health system as a critical pillar for social justice is key in addressing FGM while executing the core mandate of disease prevention and management. By leveraging opportune moments, events and experiences involving client-provider interactions, relevant FGM-related communications, behavior change and management interventions can be implemented through health facilities or in communities. It is unclear whether Kenyan health system has maximized this strategic advantage and positioning to address FGM.

**Objective:**

Determine the quality of services offered to women with FGM across health facilities in West Pokot county, Kenya.

**Methods:**

A mixed quantitative data collection strategies were used. These included: client-provider interactions observations with (61) health care workers (HCWs) and women with FGM seeking services; client-exit interviews with (360) women with FGM seeking services. These approaches sought to determine the content and quality of FGM-related care services; and service data abstractions involving records on services sought/offered from (10) facilities in West Pokot.

**Results:**

A large (76%) proportion of women had experienced FGM aged 11–15 years, were married between 15 and 19 years (39%), had primary (47.5%) or no education (33%) with income <30 USD/month (43%). Only 14.8% HCWs identified FGM and related complications (11.5%) during consultations. Few FGM-related prevention interventions were implemented with IEC materials (4.9%) for reinforcing preventive messages lacking. Infrastructure (88.5%) for reproductive health services existed albeit limited human resources (14.8%) and capacity (42.6%) for FGM prevention and management; few (16%) health facilities and workers explained the negative consequences of FGM and need for stopping it (15.3%); and while data on women who sought antenatal (ANC), postnatal (PNC) and family planning (FP) care services were available no information of those with FGM or related complications.

**Conclusion:**

Health systems in high prevalent settings actively interface with women with FGM, despite the primary reason for seeking services not being FGM. Despite high number of women having undergone the cut, diagnosis, prevention, care services, and documentation of FGM and related complications are suboptimal. This underscores the need for health system strengthening in response to the practice with consideration for training kits for HCWs, empowering HCWs, anchoring of FGM indicators in the HMIS, documentation and IEC material to support FGM prevention at service delivery points, and overall integration of FGM into health programs.

## Introduction

Continued practice of female genital mutilation (FGM) in over 90 countries globally portends perpetuation of gender inequality, negative health consequences, human rights abuse and violation, discrimination and compromise the achievement of full potential for women and girls [1, 2]. FGM is commonly practiced in 28 African countries spanning from West through Central to East and the Horn of Africa (FGM belt) as well as in the Middle East, Latin America, Asia and among the diaspora communities [1–4]. So far, it is estimated that more than 200 million girls and women have undergone some form of FGM, with over 4.1 million girls at risk of being cut every year [1]. In Kenya, the national FGM prevalence among women 15–49-year-old was 21% as of 2014 with most ethnic groups practicing it [5]. However, despite cycles of Kenya demographic health surveys (KDHS) demonstrating consistent decline in FGM, regional and ethnic variations exist with some communities in Rift Valley and North Eastern counties posting prevalence of over 90% percent [5]. This prevalence is in addition to evidence that the number of girls exposed to FGM have been exacerbated by COVID-19 pandemic-related disruptions on protection and school systems since 2020 [6–8]. Therefore, as countries prioritize COVID-19 interventions, commensurate efforts for FGM-related programs should be escalated and accelerated.

Defined as all procedures that involve partial or total removal of external female genitalia or other injuries for non-therapeutic reasons [9]. The world health organization (WHO) categorizes FGM into four types namely clitoridectomy, incision, infibulation and others all of which are practiced by various ethnic groups in Kenya [5]. FGM is associated with a number of health complications [10, 11]. These range from immediate, short and/or long-term including the immediate physical, gynecologic, obstetric, and sexual complications [9, 11]. Women and girls also suffer FGM-related psychological consequences characterized by anxiety, depression, post-traumatic stress disorder and low self-esteem [12]. The physical complications are related to the extent of cutting, performer’s poor knowledge of anatomy and/or use of crude/non-sterilized instruments during the procedure [9, 11]. The FGM-related complications require a robust health system manned by knowledgeable and skillful health care workers (HCWs) capable of responding before cutting happens through prevention or through quality care services for women who undergo the practice.

In fulfilling the mandate of prevention and management of FGM, the health system should align the interventions to the national and sector-specific legal/policy documents that are anchored on; global call for protection of human rights for women and girls articulated in the international treaties and reiterated by World Health Organization (WHO), and ratified and domesticated by member countries [13, 14]; sustainable development goals target 5.3 on eradication of harmful practices including FGM by 2030 [15]; recommendations of the treaty monitoring bodies (TMBs) for health care providers to spearhead human rights protection through FGM-related prevention and care services [9, 13]. Consistent with the progress achieved in addressing FGM at international and national levels, the WHO-led health sector FGM interventions have been commendable in providing policy leadership, developing tools to support response for FGM-related complications as well as prevention of the practice including its medicalization [9, 16–19]. These efforts and interventions should translate into FGM-related practical solutions and services provided to women and girls in the health facilities and communities where these problems are encountered.

Kenya health sector specific FGM-related interventions have gained traction at policy level with a policy statement banning medicalization issued by the minister of Health [20, 21], a training manual for health care providers developed [22], a training package for capacity building of in-service health workers as well as for trainees in medical and allied health training institutions under development courtesy of funding from WHO, UNFPA and FCDO [23]. Despite these efforts challenges persist including limited knowledge and skills for health workers to meaningfully participate in FGM prevention and care, lack of FGM related data collection at service points that can be escalated to policy makers for decision making and investment, and absence of integration of FGM into existing health programs [24]. There is minimal evidence of ongoing health sector-led FGM awareness campaigns either at facilities, in the community or through outreaches. Furthermore, there is lack of data in health facility regarding how many women present with FGM, the type of complications they present with and services offered to mitigate FGM-related complications. Related to aforementioned, it is not clear on the quality of FGM-related interventions critical for prevention or care services for women with FGM-induced complications across health facilities. This study therefore sought to determine the quality of existing FGM related services offered across health facilities in West Pokot county in Kenya.

## Material and methods

### Study design

We adopted mixed quantitative methods to determine the quality of interventions relevant to prevention and response to women and girls with FGM seeking care in health facilities within West Pokot county. To achieve the aforementioned, we conducted: (1) client-provider interactions observations to obtain data on the content and quality of FGM-related services; (2) client-exit interviews to determine the content and quality of services during consultation with HCWs; and (3) service data abstractions to review health facility records on FGM-services sought/offered (Table 1).

**Table 1:** Summary of data collection approaches with their corresponding sample size

S/N	Data gathering activity	Study Population	Sample size	Study location
1	Observations of client-provider interactions	Health care providers and women with FGM seeking reproductive health services	61	County level health facilities
2	Client-exit interviews	Women with FGM seeking reproductive health services	360	County level health facilities
3	Service data abstraction	Health facility administers, health care providers	10	County level health facilities

### Study sites

The study involved participants and respondents working and seeking services in health facilities across West Pokot county. The facilities included those categorised as primary through to tertiary referral levels. The county of West Pokot county is located in Rift Valley neighbouring the Republic of Uganda. The county covers an area of 9169.39 KM^2^. The county is resident to the Pokot people and other dominant Kalenjin communities with an estimated population of about 621,241 people as of 2019 [25]. The County had 127 health facilities categorized into; 87 Public, 3 non-governmental, 20 faith-based and 16 private operated facilities, respectively [26]. The health provider to population ratio was documented at: 67 nurses, 5 doctors and 23 clinical officers per 100,000 people [26]. As of 2014, the prevalence of FGM was at a high of 94% [5], mainly type III (infibulation), performed by traditional cutters. There is no documented evidence of health care providers involvement in cutting girls (medicalisation) in the county. The practice of FGM is performed on young girls (12 to 14 years old) as a rite of passage in preparation for adulthood, associated respect and cultural conformity. The practice of FGM is executed annually (usually during the December holidays) on all girls of appropriate age group with celebrations marked as an important community activity. There are reported cases of cross-border FGM activities because the members of Pokot community share a porous border with their counterparts from Uganda. Health wise, most women deliver at home assisted by traditional birth attendants, with only 25.8% of births delivered in health facilities [5]. Additionally, the traditional birth attendants have been reported to perform FGM on women who may have escaped the cut as girls during childbirth. The county has high maternal and infant mortality rates. Programmatically, West Pokot has been a target county for the UNFPA-UNICEF Joint Programme on accelerated abandonment of FGM.

### Study participants and respondents

The study participants and respondents included HCWs namely doctors, nurses/midwives and clinical officers who worked in service delivery points (antenatal, postnatal, family planning, delivery and new-born services, accident and emergency departments) that encountered and/or dealt with women/girls with FGM-related complications in public and private health facilities across all levels of the health system. The HCWs provided information on content, quality of services as well as data on women with FGM who sought health services. The girls (14–17 years) and women (18 years and above) who had sought services were also recruited. The women/girls with FGM provided information on the content and quality of care service offered by the health facilities.

### Sampling and sample size

A multistage sampling strategy was used to obtain appropriate sample for the participants/respondents and health facilities. We clustered the facilities into different categories (tertiary, secondary, primary and private/faith-based) using a list of health facilities obtained from the county health office. The clustering of private/mission-led facilities together was based on their small number and the challenge of categorizing using the government nomenclature. Most of the mission/private would fit as clinics while majority may not fit as the dispensaries and health Centre’s as per the government classification. We sampled all county referral and sub-county hospitals in West Pokot. Respondents were recruited for the exit interviews and client-provider interactions observations while seeking services from the sampled health facilities. Eligible girls (14–17 years) and women (18 years and older) with FGM were identified, recruited and consent and assent obtained to participate in the interviews as well as the client-provider interaction observations. Those who participated in the exit interviews and client provider interactions were purposively sampled after they met the inclusion criteria. The facilities (*n* = 61) used for client-provider interactions were distributed across the four sub-counties in West Pokot namely; Pokot Central, Pokot South, Pokot West and Pokot North. As regards data abstraction, ten (*n* = 10) facilities were sampled.

### Study instruments

The study instruments used included client-provider interactions checklist, client exit interview questionnaire and facility data abstraction checklist. These tools were adopted and modified from a handbook on “Assessing Integration Methodology”- a handbook for measuring and assessing the integration of family planning and other reproductive health Services [27].

The client-provider interaction checklist was used to capture data on the two-way interaction process between the provider and client on identification, management, and prevention of FGM. The checklist had sub-sections on availability of services to identify, manage and prevent FGM with specific statements to check whether diagnosis, management and prevention of FGM took place during the session. Additionally, the checklist had specific questions that sought to answer whether there was: private area for consultations; service provider with adequate knowledge of FGM was available; availability of different service providers to help with FGM cases such as counsellors, psychologists, medical personnel with adequate sexual and obstetric knowledge and availability of equipment needed for consultation and provision for information, education and communication (IEC) related materials to help with reinforcement of prevention messages. The checklist had questions about long term complications of FGM such as genital scarring, urinary tract complications, psychological effects and Obstetric complications.

The client exit interview questionnaire comprised of socio-demographic data, information on personal experience with FGM and health, for example, whether the client had undergone FGM and at what age, reasons why the respondent visited the health facility, as well as whether the health provider discussed about FGM-related complications with her. Furthermore, the questionnaire contained components on follow-up care, referrals, satisfaction with services, costs as well as accessibility of the health services. The questionnaire was translated into Kiswahili and in the local West Pokot languages by graduates’ research assistants from the local community and counter-checked by an independent local dialect specialist. During the training of the research assistants who were locally sourced, the tools were pretested and corrected accordingly. The term used in the translated tools was female circumcision to allow for understanding, cooperation from the local community as well as not to appear judgemental.

The health facility data abstraction checklist contained the following: facility identification, information on service statistics such as; number of pregnant women attending ANC, and number of pregnant women with FGM attending ANC. Additional questions were: number of women/girls with FGM-related complications, number of women/girls undergone FGM who received counselling as well as the number of women/girls with FGM who received antibiotics and painkillers among others. The tools were pretested and validated during research assistant trainings with feedback incorporated to refine and improve them accordingly.

### Recruitment and interview procedures

The recruitment was preceded with an exploratory visit to the county of West Pokot prior to the formal commencement of the study. A letter describing the study objectives and research authorisation permit from the national government was submitted to the county commissioner and the county government administration during the visit. Thereafter, a letter authorising the formal research involving health facilities, health workers, and clients was granted by the Director of Health Services and West Pokot County commissioner. We obtained written or verbal informed consent from all participants and respondents aged 18 years and older, while assent was obtained for respondents younger than 18 years while consenting was granted by parent/guardian or husband or male partner. However, the interviews were conducted on individual basis. The Participants were assured of confidentiality in handling the shared information, voluntary participation, need to answer only questions they were comfortable with and in case of discomfort/distress the available options to help them go through the experience. Except for the exit interviews which were conducted in local dialect, other interviews were conducted in English by trained locally recruited research assistants.

### Data analysis

Data were coded and entered into Epi-data 3.1 on password-protected computers by trained data clerks and exported to STATA version 14.2 for quality assurance and cleaning. The cleaned data were then analysed using STATA version 14.2 as explained in wider substantive report on health systems response on prevention and management of FGM in West Pokot (24). The variables were descriptively analysed into frequencies and proportions and presented mainly in tables.

### Ethical considerations

All research involving participants was performed in accordance with the Declaration of Helsinki, followed the principles and guidelines outlined in the Belmont report and were in compliance with relevant national guidelines and regulations. Ethical approval for this study was granted by the Population Council’s Institutional Review Board (Ref: 830; dated: October 16, 2017) and AMREF Health Africa Ethics and Scientific Review Committee (Ref: AMREF-ESRC P463/2018; dated: July 3, 2018). In addition, administrative approval to carry out the study was granted by National Commission for Science, Technology and Innovation (Ref: NACOSTI/P/18/79790/24356; dated: August 18, 2018), as well as the West Pokot County Commissioner and the County Director of Health Services. Study participants aged 18 years and older signed informed consent, while participants younger than 18 years provided assent to participate while informed consent was provided by a parent and/or legal guardian. Participants were informed and taken through the study protocol including measures to ensure confidentiality of the information shared and their rights to withdraw from the study at any time.

## Results

### Characteristics of health facilities utilised for client-provider interaction observations

A total of 61 client-provider interaction observations were conducted across various health facilities. The facilities were located and distributed in four sub-counties of West Pokot namely: Pokot central (39.3%), Pokot south (24.6%), Pokot West (21.3%) and Pokot North (14.8%). Most (92%) of the observations were conducted in the primary level health facilities—dispensaries and health centres.

### Identification of FGM and its related complications during client-provider interactions

The HCWs who participated in client-provider interactions attempted to identify FGM status from the respondents (Table 2). Only 15% of HCWs in primary (9.3%), secondary (45.5%) and none in tertiary health facilities asked about FGM status of the women. Further, only in 7% primary and 18.2% secondary-level facilities were respondents asked about the severity of FGM. Similarly, in less than 10% of primary and 30% secondary-level facilities, the respondents were asked about possible effects of FGM and how the practice could have undermined their physical and mental health. However, none of the HCWs at the tertiary facility asked about FGM status, its severity and the possible effects as well as physical and mental complications of the practice.

**Table 2:** Efforts to identify FGM and related complications by health workers during client-provider interactions

	Level of Facility							
	Primary ***N*** = 43		Secondary ***N*** = 11		Tertiary ***N*** = 7		Total ***N*** = 61	
	n	%	n	%	n	%	n	%
Asked client on cut FGM status								
No	38	88.4	6	54.5	7	100	51	83.6
Yes	4	9.3	5	45.5	0	0	9	14.8
Missing	1	2.3	0	0	0	0	1	1.6
Asked about the severity of cut								
No	40	93	9	81.8	7	100	56	91.8
Yes	3	7	2	18.2	0	0	5	8.2
Asked about possible effects of FGM								
No	39	90.7	8	72.7	7	100	54	88.5
Yes	4	9.3	3	27.3	0	0	7	11.5
Explain how FGM might have undermined health physically and mentally								
No	41	95.3	8	72.7	7	100	56	91.8
Yes	2	4.7	3	27.3	0	0	5	8.2

### The FGM-prevention initiatives available across health facilities in west Pokot

A number of FGM prevention interventions were implemented across health facilities as per the client-provider interaction (Table 3). Only few primary (14%), secondary (45.5%) and tertiary (14.3%) facilities, were the respondents educated or advised about available approaches for prevention of FGM by the HCWs. In about 30% of the facilities, the HCWs sensitised the respondents about availability of: linkage with authorities for reporting potential risk of FGM (32.8%), follow up mechanism (26.2%), existence of linkage with local community civil groups and gatekeepers to help in case of FGM (42.6%), and possible outreaches within the community for prevention of FGM (31.1%). However, the provision of IEC materials for reinforcing FGM prevention was very minimal (4.9%) across the facilities.

**Table 3:** Availability of FGM-prevention interventions across health facilities in West Pokot

Interventions for FGM preventions	Level of Facility							
	Primary (***N*** = 43)		Secondary (***N*** = 11)		Tertiary (***N*** = 7)		Total (***N*** = 61)	
	n	%	n	%	n	%	n	%
Educated and advised on prevention of FGM								
No	37	86	6	54.5	5	71.4	48	78.7
Yes	6	14	5	45.5	1	14.3	12	19.7
Missing	0	0	0	0	1	14.3	1	1.6
Advised on availability of linkage to authorities for reporting potential risk of FGM								
No	30	69.8	7	63.6	3	42.9	40	65.6
Yes	13	30.2	4	36.4	3	42.9	20	32.8
Missing	0	0	0	0	1	14.3	1	1.6
Existence of mechanism for follow-up								
No	31	72.1	10	90.9	2	28.6	43	70.5
Yes	11	25.6	1	9.1	4	57.1	16	26.2
Missing	1	2.3	0	0	1	14.3	2	3.3
Provision of IEC related materials to help with reinforcement of FGM prevention								
No	43	100	8	72.7	6	85.7	57	93.4
Yes	0	0	3	27.3	0	0	3	4.9
Missing	0	0	0	0	1	14.3	1	1.6
Advise on existence of link with community civil groups and gatekeepers to help in case of FGM								
No	26	60.5	4	36.4	3	42.9	33	54.1
Yes	16	37.2	7	63.6	3	42.9	26	42.6
Missing	1	2.3	0	0	1	14.3	2	3.3
Possible outreaches in community to help with prevention of FGM								
No	31	72.1	6	54.5	3	42.9	40	65.6
Yes	11	25.6	5	45.5	3	42.9	19	31.1
Missing	1	2.3	0	0	1	14.3	2	3.3

### Availability of adequate resources for FGM prevention and management response across health facilities

Health facilities and human resources for FGM prevention and management were identified during client-provider interactions (Table 4). A majority (88.5%) of health facilities had adequate space with provisions for privacy during consultation, and the appropriate equipment and service commodities for consultation (78.7%) for example stethoscope, flashlight, antiseptic solution among others. However, limited human resources and capacity for FGM response was identified. For example, only 42.6% of the facilities had adequately knowledgeable HCWs on FGM, with 14.8% of the facilities having various cadres with specialisation (e.g counsellors, psychologists, and medical personnel) for addressing FGM cases. Additionally, 63.9% of HCWs were able to explain the referral pathway for FGM cases, while 49.2% described the relationship between health system and legal authorities in addressing FGM.

**Table 4:** Availability of resources for FGM-prevention and management across health facilities

	Level of facility							
	Primary *N* = 43		Secondary *N* = 11		Tertiary *N* = 7		Total *N* = 61	
	n	%	N	%	n	%	n	%
Infrastructure								
Avails private area for consultations								
No	5	11.6	2	18.2	0	0	7	11.5
Yes	38	88.4	9	81.8	7	100	54	88.5
Service provider with adequate knowledge of FGM								
No	27	62.8	2	18.2	4	57.1	33	54.1
Yes	14	32.6	9	81.8	3	42.9	26	42.6
Missing	2	4.7	0	0	0	0	2	3.3
Availability of different service providers to help with FGM cases								
No	39	90.7	10	90.9	1	14.3	50	82
Yes	2	4.7	1	9.1	6	85.7	9	14.8
Missing	2	4.7	0	0	0	0	2	3.3
Explains the existence of referral pathway for FGM cases to higher level of care								
No	17	39.5	4	36.4	0	0	21	34.4
Yes	25	58.1	7	63.6	7	100	39	63.9
Missing	1	2.3	0	0	0	0	1	1.6
Describes the existence of association of health facility with legal authorities								
No	24	55.8	5	45.5	1	14.3	30	49.2
Yes	18	41.9	6	54.5	6	85.7	30	49.2
Missing	1	2.3	0	0	0	0	1	1.6
Avails the necessary equipment needed for consultation								
No	9	20.9	3	27.3	0	0	12	19.7
Yes	34	79.1	7	63.6	7	100	48	78.7
Missing	0	0	1	9.1	0	0	1	1.6

### Characteristics of respondents for client-exit interviews

A total of 360 exit interviews were conducted with respondents with their demographic characteristics summarised in Table 5. Thirty-nine percent were aged 15–19 years, while 38% were between 20 and 30 years. About half (53%) were in a monogamous marriage, with 52% reported having 1–3 children. Of the respondents, a third (33%) had never attended formal schooling while 48% had attained primary level education. Only a quarter (26%) reported having no income at all, 17% earned less than 3000 Kenyan Shillings (~ 30 US dollars), 16% between 3000 and 5000 Kenyan Shillings, 13% between 10,000–20,000 Kenyan Shillings, while 7% earned more than 20,000 Kenyan Shillings. Most (76%) of those interviewed reported having undergone FGM aged 11–15 years, with the procedure mainly (95%) performed by traditional circumcisers.

**Table 5:** Demographic characteristics of respondents for exit interviews

Characteristics	Facility Level							
	Primary *N* = 220		Secondary *N* = 98		Tertiary *N* = 42		Total *N* = 360	
	n	%	N	%	n	%	n	%
Age								
< 15 years	3	1.4	1	1	0	0	4	1.1
15–19 years	95	43.2	33	33.7	12	28.6	140	38.9
20–24 years	37	16.8	21	21.4	10	23.8	68	18.9
25–30 years	35	15.9	18	18.4	13	31	66	18.3
30 years or older	50	22.7	25	25.5	7	16.7	82	22.8
Marital status								
Married/monogamous	100	45.5	61	62.2	31	73.8	192	53.3
Married/polygamous	83	37.7	29	29.6	9	21.4	121	33.6
Single, never married	30	13.6	5	5.1	2	4.8	37	10.3
Divorced/separated/widowed	2	0.9	0	0	0	0	2	0.6
Missing	5	2.3	3	3.1	0	0	8	2.2
Number of living children								
None	30	13.6	8	8.2	3	7.1	41	11.4
1–3 children	108	49.1	50	51	29	69	187	51.9
4–7 children	52	23.6	23	23.5	6	14.3	81	22.5
7 or more children	26	11.8	11	11.2	3	7.1	40	11.1
Missing	4	1.8	6	6.1	1	2.4	11	3.1
Education level								
Did not attend formal school	73	33.2	35	35.7	11	26.2	119	33.1
Primary	109	49.5	48	49	14	33.3	171	47.5
Secondary	33	15	8	8.2	12	28.6	53	14.7
Tertiary	3	1.4	6	6.1	5	11.9	14	3.9
Missing	2	0.9	1	1	0	0	3	0.8
Monthly income (Kenyan Shillings)								
None	31	28.4	9	17	10	35.7	50	26.3
< 3000	25	22.9	5	9.4	2	7.1	32	16.8
3000 to < 5000	20	18.3	9	17	1	3.6	30	15.8
5000 to < 10,000	22	20.2	13	24.5	5	17.9	40	21.1
10,000 to < 20,000	8	7.3	9	17	7	25	24	12.6
20,000 or more	3	2.8	8	15.1	3	10.7	14	7.4
Total	109	100	53	100	28	100	190	100
Missing/Don’t Know	111	50.5	45	45.9	14	33.3	170	47.2
Age of undergoing FGM								
8–10 years	9	4	8	9	3	7	20	5.6
11–15 years	169	77	75	77	30	71	274	76.1
16–19 years	38	17	15	15	8	19	61	16.9
20 years and above	4	2	0	0	1	2	5	1.4

### Content and quality of consultation during reproductive health services visit by respondents

The content and quality of consultation by respondents while seeking reproductive health services was obtained through the client-exit interviews (Table 6). Most (90.8%) of the HCWs explained to the respondents the problems they were managing, provided treatment instructions (89.2%), follow up care information (76.7%), appointments for follow up visits (98.2%) and minimal referrals (3.3%) in regard to reproductive health services. However, only in 16% of facilities were respondents explained to about the negative consequences of FGM and the need to stop practicing FGM (15.3%) by the HCWs.

**Table 6:** Content and quality of information shared with respondents during reproductive health services consultations

Communication	Facility Level							
	Primary *N* = 220		Secondary *N* = 98		Tertiary *N* = 42		Total *N* = 360	
	(n)	%	(n)	%	(n)	%	(n)	%
Did the provider explain to you what he/she is managing and why?								
No	(16)	7.3	(11)	11.2	(5)	11.9	32	8.9
Yes	(204)	92.7	(86)	87.8	(37)	88.1	327	90.8
Missing	(0)	0	(1)	1	(0)	0	1	0.3
Did the provider explain the treatment instructions?								
No	23	10.5	10	10.2	5	11.9	38	10.6
Yes	196	89.1	88	89.8	37	88.1	321	89.2
Missing	1	0.5	0	0	0	0	1	0.3
Did the provider explain to you the negative consequences of FGM?								
No	180	81.8	83	85	39	93	302	84
Yes	40	18.2	15	15	3	7.1	58	16
Did the provider discuss with you the need to stop practicing FGM?								
No	181	82.3	84	85.7	39	92.9	304	84.4
Yes	38	17.3	14	14.3	3	7.1	55	15.3
Missing	1	0.5	0	0	0	0	1	0.3
During your visit today, were you given any information on follow-up care?								
No	62	28	14	14.3	8	19	84	23.3
Yes	158	72	84	85.7	34	81	276	76.7
During your visit today, were you given an appointment for a follow-up visit?								
No	2	1.3	1	1.2	2	5.7	5	1.8
Yes	157	99	84	98.8	33	94.3	274	98.2
During your visit today, were you referred for any services?								
No	205	93.2	96	98	37	88.1	338	93.9
Yes	8	3.6	1	1	3	7.1	12	3.3
Missing	7	3.2	1	1	2	4.8	10	2.8

### Data on women/girls who received reproductive health services across facilities

Data from services statistics were conducted in ten health facilities including: one tertiary, two secondary, five primary and two private/mission facilities. Data showed existence of records on reproductive health services offered across the facilities. Data on number of women/girls who sought ante-natal, post-natal care and family planning services were captured. However, data on the number of women/girls with FGM and/or related complications were lacking. There were no data on women/girls who sought or received FGM-related services and treatment such as counselling, de-infibulation, clitoral reconstruction, antibiotics for infection, pain killers, and surgical interventions for keloids, cysts, or scarring specific to FGM.

## Discussion

A summary of findings from this study shows: a large proportion of young women who had experienced FGM and early marriage had low educational attainment and income; identification of FGM and its related complications by HCWs during consultation with women/girls at the service delivery points was sub optimal; FGM-related prevention interventions were inadequate with lack of IEC materials to reinforce the preventive strategies; despite adequate infrastructure for supporting reproductive health services, it was characterized by limited human resources and capacity to implement FGM prevention and management; sensitization and awareness creation on the negative effects of FGM and the need to stop the practice existed in few health facilities implemented by few HCWs; data on clients who sought ANC, PNC and FP services existed with no information on the number of women with FGM or those with FGM-related complications.

A large proportion of young women who experienced FGM had also: married early, low educational attainment as well as low income. This is evidenced by 76% of women who reported to have been cut, aged 11–15 years with 39% having been married off aged 15–19 years. This is an indication that FGM is performed as a rite of passage perpetuating early and child marriage in West Pokot. This depicts a critical nexus between FGM and early marriage [28, 29] - practices with far reaching socio-economic impacts on women and girls, family and community. The practice of FGM is further linked with negative impacts on girl-child education with analyses showing only 48% of the women attained primary education coinciding with dropouts linked with age of FGM and the attendant early marriage in West Pokot. The findings show inadequate uptake of educational opportunities with resultant dis-empowerment narrows women choices to only marriage, housewife and child bearing responsibilities further relegating them to poverty and curtailing realization of full potential. Evidence show girls born and raised by women with no education are themselves likely to miss on educational opportunities [30, 31]. Furthermore, mothers who have undergone FGM are more likely to allow performance of the cut on their daughters perpetuating the practice and poverty through generations [32].

The practice of FGM is associated with impediment to schooling or school dropouts with the resultant meagre financial earnings or resources as evidenced by less than USD 30 per month for most (58.9%) of the women studied in West Pokot. The economic and cost burden of FGM cannot be overlooked, although WHO has developed a cost calculator and demonstrated the amount of money saved through management and prevention of direct health impacts of FGM [18], the price tag for indirect impacts such as loss of education, school dropout, lost job opportunities and poverty need to be quantified as well [33]. Indeed, the narrative of savings that could be accrued from FGM interventions should act as great incentive for policy makers to address FGM. Interestingly, support for formal education of girls has been touted as a more effective approach to FGM abandonment as schooling starves off the risk of cutting the girls and is a more girl-centered approach [31, 32]. Moreover, a combined and pairing of approaches of end FGM interventions with awareness creation and legal approach can improve their effectiveness  [36–38]. As regards the health sector, it is worth mentioning that most of the interviews were conducted in primary level facilities (health centres and dispensaries) demonstrating the importance of promotive and preventive interventions that can cost effectively be implemented through nurses, midwives, clinical officers, community health workers and volunteers at community level through integration into universal health care package.

Adequate infrastructure and human resources for supporting priority reproductive health care needs and services were identified across West Pokot. This was evidenced by the fact that health facilities and HCWs were providing antenatal, postnatal care as well as family planning services. These programs have been prioritised in the country and through donor financial investments in requisite commodity purchases, stocking, development of policies and guidelines. Furthermore, there has been capacity building and support strategy of HCWs for successful implementation and programming as well as monitoring and evaluation to track progress [39–44, 45]. This enabling policy environment is important for successful program implementation at the service delivery points and feed backing for decision making.

Financing and availing of policies and support tools as well as capacity building initiatives in response to FGM was lacking. Indeed, there was inadequate human resources in terms of requisite specialties to deal with myriad FGM-related complications as well as capacity deficits to respond to FGM prevention and management. The services relevant to FGM prevention and care services were characterised by: few HCWs who identified FGM and related complications during consultation with women and girls; few FGM-related prevention interventions that were implemented; lack of IEC materials to reinforce the FGM preventive messages; and few health facilities and HCWs who offered explanation on the negative consequences of FGM and the need to stop the practice. These findings are consistent with evidence that FGM has not been receiving the required attention in the health sector because of competing priorities in Kenya and elsewhere including Australia [46–48]. However, recently in line with the global agenda direction, Kenya health sector specific FGM-related interventions have gained traction at policy level with a policy statement banning medicalization issued by the minister [20, 21], a training manual for health care providers developed [22], a training package for capacity building of in-service health workers as well as for trainees in medical and allied health training institutions under development courtesy of funding from WHO, FCDO and UNFPA [23]. It is hoped that the enabling legal-policy environment, availability of tools to support training and capacity building will catalyze health sector-led practical solutions in response to FGM prevention and management. This will be complemented with the ongoing agenda for integration of FGM interventions into existing health services in the service points that interface with women with FGM to leverage on their success and available resources.

We show evidence on availability of data on women who sought antenatal, postnatal and family planning services with no information on those with FGM or related complications. The absence of documentation is attributed to lack of FGM-related data capture tools at the service delivery points as well absence of FGM indicators in the health management information system (HMIS). Anchoring of FGM indicators in the HMIS will prompt the development of FGM data collection tools for use in the service delivery points. This process has already commenced with FGM indicators having been developed, while the training tools are underway through the support of WHO. This will trigger training of the health care providers on FGM, data acquisition, dissemination and use of the tools. The development of the FGM data capture tools should leverage on similar tools such as the post-rape care (PRC) form used in gender-based violence case management. This may be facilitated through deliberate integration of FGM into existing health programs as well as monitoring and evaluation pathways.

The findings should be interpreted bearing in mind some limitations namely; the study was conducted in only one county of West Pokot where type III FGM is predominant limiting generalizability to the whole country; the observation of provider-client interaction portends that there could have been some performance bias from the participants knowing they were being rated; this is a facility based data that is biased towards to those who sought health services and may not be generalizable to all women in West Pokot; the study data are from a cross-sectional study with limitation that, it precludes our ability to make causal inferences; The participants and respondents were not randomly sampled introducing some selection bias. However, our approach was premised on multiple data methods that adds to the confidence in the results.

## Conclusion

In conclusion, the health systems in FGM high prevalent settings are actively interfacing with women with FGM, despite their primary reason for health seeking not being FGM. Despite the high number of women with FGM, identification of the problem, prevention and response to FGM complications as well as documentation of the cases and related complications in the service delivery points are suboptimal. This underscores the need for health system strengthening in response to FGM that should consider development of training kits for HCWs, training of HCWs, anchoring of FGM indicators in the HMIS, documentation, and development of IEC materials to support FGM response at service delivery points, and overall integration of FGM into ongoing health programs.

## Data Availability

The datasets generated and/or analysed during the current study are not publicly available due to the data protection policy of University of Nairobi but are available from the corresponding author on reasonable request.

## Abbreviations

ANC: Antenatal Care
FCDO: Foreign, Commonwealth & Development Office
FGM: Female Genital Mutilation
FP: Family Planning
HCWs: Health care providers
HMIS: Health Management Information System
PNC: Post Natal Care
UNFPA: United Nations Population Fund
WHO: World Health Organisation
UN: United Nation
